# Pericardial Mesothelioma: Diagnostic and Therapeutic Management, a Population-Based Study in Italy

**DOI:** 10.3390/cancers17233865

**Published:** 2025-12-01

**Authors:** Simona Stella, Dario Consonni, Giovanni Luca Ceresoli, Barbara Dallari, Riccardo Perduri, Cinzia Storchi, Enrica Migliore, Manuela Gangemi, Carlo Genova, Lucia Benfatto, Vera Comiati, Valentina Zabeo, Sara Piro, Lucia Giovannetti, Iolanda Grappasonni, Cristiana Pascucci, Francesca Larese Filon, Flavia D’Agostin, Luigi Vimercati, Ilaria Cozzi, Franco Calista, Giuseppe Cascone, Italo Francesco Angelillo, Alessandra Binazzi, Alessandro Marinaccio, Carolina Mensi

**Affiliations:** 1 COR Lombardia, Occupational Health Unit, Fondazione IRCCS Ca’ Granda Ospedale Maggiore Policlinico, 20122 Milan, Italy; simona.stella@policlinico.mi.it (S.S.); dario.consonni@unimi.it (D.C.); barbara.dallari@policlinico.mi.it (B.D.); 2 Department of Medical Oncology, Humanitas Gavazzeni Clinic, 24125 Bergamo, Italy; giovanniluca.ceresoli@gmail.com; 3 COR Mesoteliomi Emilia Romagna, AUSL-IRCCS di Reggio Emilia, 42123 Reggio Emilia, Italy; riccardo.perduri@ausl.re.it (R.P.); cinzia.storchi@ausl.re.it (C.S.); 4 COR Piemonte, AOU Città della Salute e della Scienza di Torino and University of Turin, 10124 Torino, Italy; enrica.migliore@cpo.it (E.M.); manuela.gangemi@cpo.it (M.G.); 5 COR Liguria, Clinical Epidemiology Unit, IRCCS Ospedale Policlinico San Martino, Dipartimento di Medicina Interna e Specialità Mediche, Università degli Studi di Genova, 16132 Genova, Italy; carlo.genova@hsanmartino.it (C.G.); lucia.benfatto@hsanmartino.it (L.B.); 6 COR Veneto, Azienda Zero, 35131 Padova, Italy; vera.comiati@azero.veneto.it (V.C.); valentina.zabeo@azero.veneto.it (V.Z.); 7 COR Toscana, Cancer Risk Factors and Lifestyle Epidemiology Unit, Institute for Cancer Research, Prevention and Clinical Network (ISPRO), 50139 Firenze, Italy; s.piro@ispro.toscana.it (S.P.); l.giovannetti@ispro.toscana.it (L.G.); 8 COR Marche, School of Medicinal and Health Products Sciences, University of Camerino, 62032 Camerino, Italy; iolanda.grappasonni@unicam.it (I.G.); cristiana.pascucci@unicam.it (C.P.); 9 COR Friuli Venezia Giulia, Clinical Unit of Occupational Medicine, University of Trieste, 34127 Trieste, Italy; larese@units.it (F.L.F.); fladagostin@yahoo.it (F.D.); 10 COR Puglia, Section of Occupational Medicine ‘B Ramazzini’, Department of Interdisciplinary Medicine, University of Bari Aldo Moro, 70121 Bari, Italy; luigi.vimercati@uniba.it; 11 COR Lazio, Department of Epidemiology, Lazio Regional Health Service, ASL Roma 1, 00139 Rome, Italy; i.cozzi@deplazio.it; 12 COR Molise, Molise Regional Health Authority Campobasso, 86100 Campobasso, Italy; franco.calista@asrem.molise.it; 13 COR Sicilia, Cancer Registry ASP Ragusa and Sicily Regional Epidemiological Observatory, 97100 Ragusa, Italy; giuseppe.cascone@asp.rg.it; 14 COR Campania, Dipartimento di Medicina Sperimentale, Università degli Studi della Campania “Luigi Vanvitelli”, 80138 Napoli, Italy; italofrancesco.angelillo@unicampania.it; 15 DIMEILA, Department of Occupational and Environmental Medicine, Epidemiology, Hygiene, National Institute for Insurance against Accidents at Work (INAIL), 00144 Rome, Italy; a.binazzi@inail.it (A.B.); a.marinaccio@inail.it (A.M.)

**Keywords:** pericardial mesothelioma, asbestos, treatment, survival, cancer registry

## Abstract

Background: Pericardial mesothelioma (PM) is an extremely rare cancer with a poor prognosis and no consensus on diagnostic and therapeutic management. We conducted a registry-based study on PM cases in Italy diagnosed between 1993 and 2021. Methods: Based on data from the Italian National Mesothelioma Registry (ReNaM), we performed a descriptive analysis of PM cases including clinical presentation, diagnostic work-up, asbestos exposure, and therapeutic management. Overall survival was calculated. Hazard ratios (HRs) and 95% confidence intervals (CI) for selected variables were estimated with univariate and multivariate Cox models. Results: We identified 72 cases (46 men and 26 women). Median age was 66 years (range 22–89). The most frequent histological subtypes were epithelioid and unspecified mesothelioma. Almost two-thirds of cases had been exposed to asbestos. The most common clinical presentation was pericardial effusion. Overall median survival was 2.8 months (95% CI 1.2–6.6) and older age at diagnosis was a negative prognostic factor. Clinical and treatment data were available for 47 patients (65%). Approximately one out of two patients underwent surgery (palliative, in one-third of cases). Adjuvant therapy was administered to seven patients (15%). In cases with treatment information, sarcomatoid subtype (HR 2.74, 95% CI: 1.06–7.06) was a negative prognostic factor; adjuvant therapy was associated with better survival (HR 0.38, 95% CI 0.14–1.02), but confounding by indication cannot be excluded. Conclusions: We confirmed the very poor prognosis of PM. An international network collecting PM cases with treatment information is needed to improve patient quality of life and survival.

## Simple Summary

Pericardial mesothelioma (PM) is an extremely rare tumour for which only case reports, small case series, and case reviews have been published. This is a large population-based study on PM in Italy using data from the Italian National Mesothelioma Registry (Registro Nazionale Mesoteliomi, ReNaM). Information collected includes detailed clinical data on diagnosis and treatment and asbestos exposure. This study confirmed the extreme rarity of PM with crude incidence rates for men and women in 1993–2021 of 0.60 and 0.30 per 10 million person-years, respectively. Epidemiological characteristics of the tumour included late age at onset, male predominance, frequent occupational asbestos exposure, and an extremely poor median survival of 2.8 months. Surgery, when feasible, is the mainstay of treatment. The role of adjuvant treatments is unclear. An early diagnosis combined with standard treatment guidelines could improve patient prognosis and quality of life.

## 1. Introduction 

Pericardial mesothelioma (PM) is an extremely rare cancer accounting for 1–2% of all mesotheliomas [1] and 4% of heart and pericardial tumours [2]. This malignancy arises from pericardial mesothelial cells and affects mostly males, with a gender ratio (male versus female) ranging from 1:1 to 3:1 [3,4,5,6]. In a recent Italian study [7], the gender ratio for PM was 1.95, increasing from 1.5 in 1993–2003 to 2.6 in 2004–2015. The Surveillance, Epidemiology, and End Results (SEER) Program from the US National Cancer Institute calculated mean annual standardised incidence rates for pericardial mesothelioma of 0.35 and 0.36 per 10 million person-years in men and in women, respectively [7].

Asbestos exposure is the primary risk factor: a recent case–control study in Italy estimated a fourfold increased PM risk for occupational asbestos exposure [7].

PM symptoms include fatigue, shortness of breath, chest pain, cough (frequently non-productive), and peripheral oedema; in fact, the clinical onset is generally related to signs of constrictive pericarditis, pericardial effusion, cardiac tamponade, and/or heart failure [5,8]. Other clinical unusual presentations are myocardial infarction, peripheral lymphadenopathy, and superior vena cava thrombosis [9,10,11].

PM survival is poor, typically between 6 and 18 months; moreover, up to 12.6% of cases are diagnosed after death [5]. Metastases occur in 25% to 45% of patients, and involve mostly regional lymph nodes, lungs, kidneys, and liver [8]. There is no consensus for disease management. Surgery seems to be associated with survival benefit in selected cases. Platinum-based chemotherapy showed increased survival in several reported case series [3,5,8]. Recently, a longer overall survival was observed in a few patients treated with trimodality therapy consisting of surgical resection, adjuvant chemotherapy, and sequential adjuvant intensity-modulated radiation therapy [12].

## 2. Materials and Methods 

### 2.1. Study Design, Participants, and Data Sources 

The Italian National Mesothelioma Registry (Registro Nazionale Mesoteliomi, ReNaM) is a population-based registry collecting information on patients with mesothelioma, at any site: pleura, peritoneum, pericardium, and tunica vaginalis testis [13]. The Registry is organised as a network of 21 regional centres (Centri Operativi Regionali, COR). In some regions, mesothelioma registration started in 1993; others began later [13].

Although compulsory by law (277/1991 and 81/2008), report of mesothelioma cases to CORs is largely incomplete [13]; therefore, CORs exploit several resources to complete case collection, including hospital discharge records and data from local health units, pathology departments, the Italian Workers’ Compensation Authority (INAIL), and mortality databases. 

For each case, the CORs collects the date of diagnosis and histological subtype (epithelioid, biphasic, sarcomatoid). For confirmed cases, qualified personnel administer a standardised questionnaire to patients or their next of kin to investigate asbestos exposure (lifetime occupational and non-occupational). Asbestos exposure is then evaluated and classified according to ReNaM guidelines as occupational or non-occupational (including familial, environmental, and home-related) [13].

### 2.2. Variables and Outcomes 

For all cases, we ascertained vital status and cause of death information as of 31 December 2023. We collected additional clinical information such as clinical presentation, symptoms, and the presence of distant metastases at diagnosis. Therapeutic management information, including the type of surgical intervention and adjuvant therapies (chemotherapy and/or loco-regional radiotherapy), was recorded.

### 2.3. Statistical Analysis 

A descriptive analysis of patient characteristics was performed. We calculated crude and standardised rates for the period 2000–2021 using the European (2013) and world (Segi’s) standard populations. A Kaplan–Meier analysis was performed to calculate overall survival. Univariate and multivariate Cox models were used to estimate hazard ratios (HR) and 95% confidence intervals (CI) for selected variables, including age at diagnosis (<45, 45–64, 65–74, and ≥75 years), histological subtype, period of diagnosis (1993–1998, 1999–2004, 2005–2010, 2011–2016, 2017–2021), presence of distant metastasis at diagnosis (no/yes), and treatment (surgery only, surgery plus any adjuvant therapy). Statistical analyses were performed using Stata Version 19 [14].

## 3. Results 

The ReNaM recorded 72 cases of PM between January 1993 and December 2021, 47 (65%) of which had additional information on clinical presentation and treatment. The crude incidence rates per 10 million person-years were 0.60 and 0.30 in men and in women, respectively. In men, age-standardised rates based on European and world standard population were 0.60 and 0.30, respectively. In women, both standardised rates were 0.30. The gender ratio (male vs. female) was 1.8 and median age at diagnosis was 66 years (range 22–89), with 60% of cases aged 65 years or older (Table 1). The most frequent histological subtype was epithelioid; one-third of cases were unspecified mesotheliomas.

Interviews were obtained for three-quarters of cases, the majority of which were indirect (56%). Among the 55 cases with available interview data, 32 (58%) had a history of occupational asbestos exposure, with men more frequently exposed (27/35, 77%) than women (5/20, 25%).

In 41 (87%) out of 47 cases with treatment information, the most common clinical presentations were pericardial effusion, heart failure/pericardial tamponade, and pericarditis or pleural effusion (Table 2). The most frequent reported symptoms were dyspnoea, malaise or fever, and chest pain. Metastases at diagnosis were present in five cases (11%).

Most cases were investigated using a contrast-enhanced computed tomography (CT) scan (Table 3). Pre-surgical cytological and histological diagnoses were obtained in a few cases, five (11%) and three (6%), respectively. In seven (15%) cases, diagnosis was performed at autopsy.

Twenty-three patients (49%) underwent surgery, with a major procedure (pericardiectomy or mass resection) in fifteen cases only (32%) (Table 4). Seven patients (15%) received adjuvant treatments: chemotherapy or, in one case, chemotherapy plus radiotherapy. The remaining patients received no treatment or palliative procedures only.

**Figure 1:**
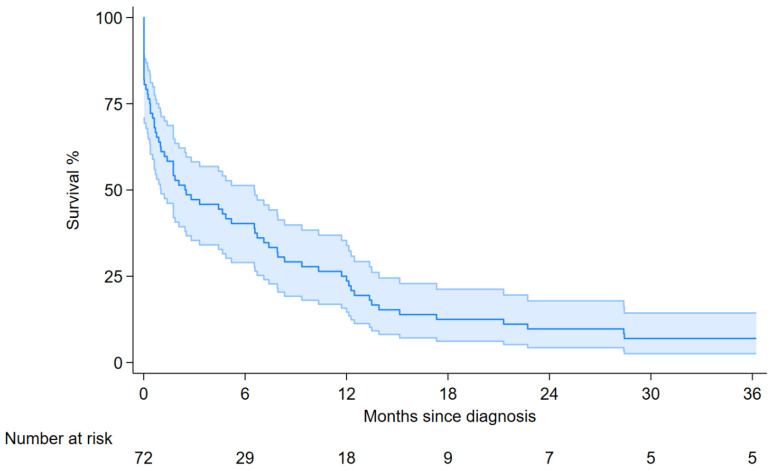
Three-year overall survival for 72 patients with pericardial mesothelioma, Italy, 1993–2021. Dark blue line: point estimates; light blue shade: 95% confidence bands.

**Table 5:** Overall survival and hazard ratios (HRs) of deaths according to selected risk factors for pericardial mesothelioma cases, Italy, 1993–2021.

Variable	N	Deaths N (%)	Overall Survival, (Months) Median	Crude HR	95% CI
**Overall**	72	67 (93)	2.8 (1.2–6.6)		
**Gender**					
Female	26	25 (96)	6.7 (4.4–12)	1.00	Reference
Male	46	42 (91)	1.2 (0.5–2.8)	1.31	0.79–2.15
**Age at diagnosis (years)**					
<45	8	7 (88)	8.3 (0.2–12.3)	1.00	Reference
45–64	21	21 (100)	6.5 (1.9–13.4)	1.37	0.57–3.26
65–74	25	23 (92)	2.1 (0.5–9.4)	1.47	0.63–3.44
≥ 75	18	16 (89)	0.7 (0.0–1.8)	2.76	1.10–6.92
**Period of diagnosis**					
1993–1998	12	12 (100)	0.6 (0.0–10.3)	1.00	Reference
1999–2004	20	20 (100)	1.9 (0.4–13.4)	0.53	0.25–1.12
2005–2010	17	16 (94)	7.1 (0.9–21.3)	0.40	0.18–0.87
2011–2016	15	15 (100)	1.8 (0.0–4.7)	0.92	0.43–1.98
2017–2021	8	4 (50)	6.5 (0.4–NC)	0.34	0.11–1.05
**Morphology (ICD-O-3 code)**					
Epithelioid (90523)	26	22 (85)	2.8 (0.5–12)	1.00	Reference
Biphasic (90533)	9	9 (100)	8.3 (0.0–22.7)	0.94	0.43–2.05
Sarcomatoid (90513)	10	10 (100)	1.2 (0.0–6.5)	2.03	0.94–4.37
Not otherwise specified (90503)	24	23 (96)	3.3 (0.7–11.7)	0.99	0.55–1.79
Not available	3	3 (100)	0.9 (0.4–NC)	2.72	0.79–9.39

Abbreviations: CI = confidence intervals; ICD-O-3, International Classification of Diseases for Oncology, Third Edition. NC = not calculable.

Overall survival was 2.8 months (95% CI 1.2–6.6). In cases with treatment information, median overall survival was 1.8 months (Table 6). The prognostic role of older age (over 75 years) was confirmed, with an HR of 4.59 (95% CI: 1.38–15.24). Sarcomatoid histology showed an HR of 2.74 (95% CI: 1.06–7.06). Conversely, the administration of adjuvant therapy was associated with a reduced risk of death, with an HR of 0.38 (95% CI: 0.14–1.02). Multivariate analysis confirmed the prognostic role of older age. 

## 4. Discussion 

In this study covering the whole Italian population over a long period (1993–2021), we found 72 cases of PM, mostly men with a history of asbestos exposure, and old age at diagnosis. Overall median survival was very poor (less than 3 months) and is consistent with previous population-based studies [3]. Older age and sarcomatoid histological subtype were negative prognostic factors. Adjuvant therapy was associated with better survival, although confounding by indication (i.e., indication of adjuvant therapy only for a few patients sufficiently healthy, thereby rendering the observed survival benefit potentially attributable to patient selection rather than the treatment itself) cannot be excluded [15].

The major strengths of this study are that the case series comes from a population-based registry (not a potentially selected hospital-based series) and the large (for this very rare disease) sample size: to our knowledge, there are no other population-based studies with larger numbers than ours. The main limitation is that we were only able to retrieve complete clinical information for 47/72 (65%) patients. This presents a potential risk of selection and constrains the applicability of the prognostic findings to the broader PM population.

The age at onset of PM is highly variable, with cases reported between the second and eighth decades of life [2]; however, the majority occur between the fourth and seventh decades [16]. Our findings are consistent with this distribution, although we observed a notable subset of patients younger than 45 years.

As previously reported [5], a male predominance was observed, with a nearly 2:1 male-to-female ratio. Due to the disease’s poor prognosis, exposure assessment was based primarily on indirect interviews, which are less informative because next of kin may not know the details of occupational or non-occupational asbestos exposure. This aspect may lead to an underestimation of cases attributable to asbestos exposure. In our case series, most patients (especially men) were occupationally exposed to asbestos. 

The most common histologic subtypes were epithelioid and mesothelioma not otherwise specified (NOS). Diagnosis is often challenging due to unspecific symptoms at onset [8]. PM typically presents with dyspnoea related to pericardial effusion, thickening of the pericardium, which leads to increased intrapericardial pressure, direct myocardial infiltration by the tumour with impairment of both diastolic and systolic function, and pulmonary congestion. Pleural effusion may exacerbate dyspnoea [17,18]. Imaging combining different modalities such as echocardiography, CT scan, positron emission tomography (PET), and cardiac magnetic resonance imaging (MRI) is essential for diagnostic evaluation and classically reveals a thickened pericardium [8,18]. In our case series, nearly half of patients presented with dyspnoea, while 25% had pleural effusion. Most patients underwent two or more imaging examinations.

Differential diagnosis should be actively pursued through histopathologic examinations in patients with recurrent pericardial effusion, pericardial tamponade, progressive pericarditis, or unresponsive to anti-tuberculous and anti-inflammatory treatments [17]. However, preoperative diagnosis is uncommon in PM patients, with 10%-30% of cases only diagnosed by pericardiocentesis [5,19]. In our study, only a few cases had a cytological or histological pre-surgical diagnosis. A small proportion of cases was diagnosed via autopsy, confirming the findings of previous studies [5]. The reduced survival also affects the possibility of collecting information regarding asbestos exposure by speaking directly with the patient.

Therapeutic management for PM includes surgery, radiation, and chemotherapy. No standard treatment protocol has been established internationally [17]. Due to its dramatic clinical course, PM is often diagnosed at advanced stages [20] or post-mortem [5], although the post-mortem diagnosis rate has decreased over the years, from 75% to 13% [5]. Unfortunately, the safe, complete removal of the tumour is not possible in many cases [19]. Surgical approaches therefore include major procedures (pericardiectomy or mass resection) and palliative procedures (therapeutic pericardiocentesis and pericardial window). In our case series, only half of patients underwent surgery, and a significant proportion of these procedures were intentionally palliative.

Due to the rarity of PM, clinical trials evaluating systemic therapy and radiotherapy are lacking [17]. Patients are often treated with palliative platinum-based chemotherapy [8,18]. A recent review indicates that patients receiving platinum-based chemotherapy, with or without pemetrexed, had a better prognosis and survival [5]. The effectiveness of chemotherapy is confirmed by several case reports [17]. Additionally, evidence suggests that a multimodality approach (combining surgery, radiotherapy, and chemotherapy) may further improve prognosis [12]. In our study, 7 of 15 patients who underwent major surgery also received adjuvant chemotherapy (mostly cisplatin or carboplatin combined with pemetrexed) and this therapy group showed an improved survival. This finding is consistent with previous studies [5]. However, it should be interpreted with caution, because, as noted above, the observed survival benefit of adjuvant therapy may be attributable to confounding by indication.

In our series, older age and sarcomatoid histotype were related to a worse survival. Although metastases at diagnosis have been associated with reduced survival in other series [5], we did not find evidence supporting this association in our study. This finding could be influenced by the small number of patients with metastasis and/or incomplete information (the dramatic course of PM may have hampered a full staging procedure in a few cases). Despite the decreased rate of post-mortem diagnosis and the availability of multimodal therapeutic approaches, the prognosis of PM remains extremely poor, mainly due to the late diagnosis and the aggressiveness of the disease. An early diagnosis is of paramount importance to increase the likelihood of performing a major surgical procedure. Pericardial mesothelioma should be differentiated from benign pericardial disease and from other primary and secondary neoplasms of the pericardium [8,21]. Among emerging therapeutic perspectives, immunotherapy with nivolumab and ipilimumab, commonly used in pleural mesothelioma management, has not been reported so far in PM patients [22,23]. The administration of immunotherapy in pleural mesothelioma with pericardial involvement has been documented in isolated case reports [16,24].

## 5. Conclusions 

In conclusion, we confirmed that PM is an extremely rare and aggressive cancer, with a poor prognosis. Due to its rarity, no standardised diagnostic and therapeutic guidelines have been currently established. Early detection is crucial for enhancing the chances of a successful major surgical intervention. On the other hand, future investigations should examine emerging therapeutic approaches such as immunotherapy in this context. Further efforts, including the implementation of an international network collecting PM cases with diagnosis and treatment data, are needed to improve both patient quality of life and survival. 

## Figures and Tables

**Table 1:** Characteristics of patients with pericardial mesothelioma, Italy, 1993–2021.

Variable	N (%)	Cases with Treatment Information N (%)	Cases Without Treatment Information N (%)	*p*-Value ^a^
**Total**	72	47	25	
**Gender**				
Males	46 (64)	31 (66)	15 (60)	0.62
Females	26 (36)	16 (34)	10 (40)	
**Age, median (range)**	66 (22–89)	68 (22–85)	65 (34–89)	0.42
**Age classes (years)**				
<45	8 (11)	5 (11)	3 (12)	0.78
45–64	21 (29)	12 (26)	9 (36)	
65–74	25 (35)	17 (36)	8 (32)	
≥ 75	18 (25)	13 (27)	5 (20)	
**Period of diagnosis**				
1993–1998	12 (17)	9 (19)	3 (12)	0.09
1999–2004	20 (28)	14 (30)	6 (24)	
2005–2010	17 (24)	10 (21)	7 (28)	
2011–2016	15 (21)	12 (26)	3 (12)	
2017–2021	8 (11)	2 (4)	6 (24)	
**Morphology (ICD-O-3 code)**				
Epithelioid (90523)	26 (36)	16 (34)	10 (40)	0.56
Biphasic (90533)	9 (13)	5 (11)	4 (16)	
Sarcomatoid (90513)	10 (14)	7 (15)	3 (12)	
Not otherwise specified (90503)	24 (33)	18 (38)	6 (24)	
Not available	3 (4)	1 (2)	2 (8)	
**Exposure evaluation**				
Direct interview	15 (21)	5 (11)	10 (40)	0.26
Indirect interview	40 (56)	34 (72)	6 (24)	
None	17 (24)	8 (17)	9 (36)	
**Sources of asbestos exposure (only for cases with exposure assessment)**				
Occupational	32 (58)	24 (62)	8 (50)	0.65
Non-occupational	2 (4)	1 (3)	1 (6)	
Unexposed	21 (38)	14 (36)	7 (44)	

^a^ From Wilcoxon (Mann–Whitney) test or Chi-Squared test. Abbreviations: ICD-O-3, International Classification of Diseases for Oncology, Third Edition.

**Table 2:** Clinical and radiological presentation of pericardial mesothelioma in 47 cases with additional information on clinical presentation and treatment, Italy, 1993–2021.

Variable	N	%
**Total**	47	
**Age, median (range)**	68 (22–85)	
**Clinical presentation**	41	87
Pericardial effusion	30	64
Heart failure/pericardial tamponade	17	36
Pericarditis	11	23
Pleural effusion	11	23
Peripheral lymphadenopathy	8	17
Cardiac arrhythmia	7	15
Pericardial/mediastinal mass	5	11
Ascitic effusion	5	11
Other acute cardiovascular events	4	9
Pneumonia	4	9
Peripheral oedema	2	4
Parapericardial hypoventilative striae	1	2
**Symptoms**		
Dyspnoea	23	49
Malaise/fever	13	28
Chest pain	11	23
Cough	10	21
Asthenia	9	19
Gastrointestinal symptoms	9	19
Cardiac symptoms	4	9
**Metastases at diagnosis ^a^**		
No	42	89
Yes	5	11

^a^ lung (N = 2), brain (N = 1), other (N = 2).

**Table 3:** Diagnostic workup of pericardial mesothelioma in 47 cases with additional information on clinical presentation and treatment, Italy, 1993–2021.

Variable	N	%
**Imaging** ^a^	41	87
Chest X-rays	30	64
Contrast-enhanced computed tomography scan	37	79
Magnetic resonance imaging	12	26
Positron emission tomography	7	15
Scintigraphy	1	2
**Pre-surgical cyto-histological diagnosis**		
Cytological examination	5	11
Histological examination	3	6
**Diagnosis via autopsy**	7	15

^a^ patients may undergo two or more imaging evaluations.

**Table 4:** Upfront therapeutic management of pericardial mesothelioma in 47 cases with additional information on clinical presentation and treatment, Italy, 1993–2021.

Variable	N	%
**Total**	47	100
**Surgery**	23	49
Major surgical procedures (pericardiectomy/mass resection)	15	32
Palliative procedures (therapeutic pericardiocentesis/pericardial window)	8	17
**Surgery + adjuvant treatment(s)**		
Adjuvant chemotherapy	7	15
Adjuvant chemotherapy and radiotherapy	1	2

Median overall survival was 2.8 months (95% CI 1.2–6.6, Figure 1 and Table 5). Older age (over 75 years) was associated with a worse prognosis, with an HR of 2.76 (95% CI: 1.10–6.92).

**Table 6:** Overall survival and hazard ratios (HRs) of deaths according to selected risk factors for pericardial mesothelioma in cases with additional information on clinical presentation and treatment (N = 47), Italy, 1993–2021.

Variable	N	Deaths N (%)	Overall Survival, (Months) Median	Crude HR	95% CI
**Overall**	47	45 (96)	1.8 (0.6–4.9)		
**Gender**					
Female	16	16 (100)	6.6 (0.8–9.4)	1.00	Reference
Male	31	29 (94)	1.0 (0.3–1.8)	1.48	0.80–2.74
**Age at diagnosis (years)**					
<45	5	4 (80)	8.3 (0.8–NC)	1.00	Reference
45–64	12	12 (100)	4.9 (0.0–13.4)	2.11	0.67–6.67
65–74	17	17 (100)	1.8 (0.3–7.1)	2.18	0.73–6.54
≥ 75	13	12 (92)	0.6 (0.0–1.8)	4.59	1.38–15.24
**Period of diagnosis**					
1993–1998	9	9 (100)	0.6 (0.0–6.6)	1.00	Reference
1999–2004	14	14 (100)	1.0 (0.0–13.5)	0.48	0.20–1.15
2005–2010	10	9 (90)	1.2 (0.0–7.9)	0.53	0.21–1.38
2011–2016	12	12 (100)	1.4 (0.0–5.2)	0.93	0.39–2.21
2017–2021	2	1 (50)	4.4 (4.4–NC)	0.31	0.04–2.44
**Morphology (ICD-O-3 code)**					
Epithelioid (90523)	16	14 (88)	1.8 (0.3–9.4)	1.00	Reference
Biphasic (90533)	5	5 (100)	7.1 (0.0–NC)	1.15	0.41–3.23
Sarcomatoid (90513)	7	7 (100)	0.1 (0.0–1.4)	2.74	1.06–7.06
Not otherwise specified (90503)	18	18 (100)	1.8 (0.6–6.6)	1.12	0.55–2.25
Not available	1	1 (100)	-	2.71	0.34–21.47
**Metastases at diagnosis**					
No	42	40 (95)	1.4 (0.6–4.4)	1.00	Reference
Yes	5	5 (100)	6.6 (0.0–NC)	1.14	0.44–2.93
**Treatment group**					
No surgery	24	24 (100)	0.9 (0.0–1.8)	1.00	Reference
Palliative surgery	8	8 (100)	1.2 (0.0–8.3)	0.83	0.37–1.86
Surgery only	8	8 (100)	2.1 (0.0–7.1)	0.77	0.34–1.75
Surgery plus adjuvant therapy	7	5 (71)	7.9 (0.8–NC)	0.38	0.14–1.02

## Data Availability

Data presented in this study are available on request from the corresponding author.
